# Effects of novel non-thermal atmospheric plasma treatment of titanium on physical and biological improvements and in vivo osseointegration in rats

**DOI:** 10.1038/s41598-020-67678-z

**Published:** 2020-06-30

**Authors:** Zheng Zheng, Xiaogang Ao, Peng Xie, Jie Wu, Yuqing Dong, Deping Yu, Jian Wang, Zhimin Zhu, Hockin H. K. Xu, Wenchuan Chen

**Affiliations:** 1 State Key Laboratory of Oral Diseases and National Clinical Research Center for Oral Diseases, West China Hospital of Stomatology, Sichuan University, Chengdu, China; 2 Department of Oral Prosthodontics, West China Hospital of Stomatology, Sichuan University, Chengdu, Sichuan China; 3 School of Mechanical Engineering, Sichuan University, Chengdu, China; 4 Biomaterials and Tissue Engineering Division, Department of Advanced Oral Sciences and Therapeutics, University of Maryland Dental School, Baltimore, MD 21201 USA; 5 Center for Stem Cell Biology and Regenerative Medicine, University of Maryland School of Medicine, Baltimore, MD 21201 USA; 6 University of Maryland Marlene and Stewart Greenebaum Cancer Center, University of Maryland School of Medicine, Baltimore, MD 21201 USA

**Keywords:** Biological techniques, Biotechnology, Cell biology, Molecular biology, Zoology

## Abstract

Titanium (Ti) has achieved extensive applications due to its excellent biocompatibility and mechanical properties. Plasma can enhance surface hydrophilia of Ti with decreased carbon contamination. The traditional conditions using a single gas plasma was for longer treatment time and more prone to being contaminated. We designed and developed novel and universal apparatus and methods with a special clamping device of non-thermal atmospheric plasma (NTAP) treatment using mixed gas for Ti surface activation. We systematically and quantitatively investigated the effective effects of NTAP-Ti. The surface water contact angle decreased by 100%, the carbon content decreased by 80% and oxygen content increased by 50% in the novel NTAP-Ti surfaces. NTAP treatment accelerated the attachment, spread, proliferation, osteogenic differentiation and mineralization of MC3T3-E1 mouse preosteoblasts in vitro. The percentage of bone-to-implant contact increased by 25–40%, and the osteoclasts and bone resorption were suppressed by 50% in NTAP-Ti in vivo. In conclusion, NTAP-Ti substantially enhanced the physical and biological effects and integration with bone. The novel and universal apparatus and methods with a special clamping device using gas mixtures are promising for implant activation by swiftly and effectively changing the Ti surface to a hydrophilic one to enhance dental and orthopedic applications.

## Introduction

Biomaterials are increasingly used to repair and replace tissue and organ structures and functions^1^. Among them, titanium (Ti) and Ti alloy implants are frequently used in dental and orthopedic applications^2,3^. A major advantage of Ti is that it can establish a directly structural and functional connection between living bone and the surface of a load-bearing artificial implant, which is termed osseointegration^4^. The surface characteristics in topography, structure, chemistry, surface charge, and wettability of the implants are important in their bioreaction and are crucial for their success in implantation^5,6^. However, Ti surfaces undergo a reduction in bioactivity in ambient atmosphere due to carbon contamination, namely aging, leading to changes in surface characteristics including surface energy and wettability. Indeed, functional drawbacks in conventional implant surfaces are related to adverse surface chemistry induced hydrophobicity and contaminations, which further reduce the biologically available clean and bioactive implant surface areas^7,8^.

A hydrophilic Ti surface can be obtained by an extensive hydroxylation/hydration of the oxide layer, resulting in an interaction between the surface and the water shell around the biomolecules^9^. Previous studies demonstrated that hydrophilic Ti implants promoted protein adsorption and improved the initial blood contact on hard and soft tissue integration with the implant, thereby accelerating the osseointegration process and shortening the healing time^10,11^. Hydrophilic implants showed high success rates in irradiated, diabetes mellitus and osteoporosis patients^12–14^. Various technologies are available to prepare hydrophilic implants. Ti and Ti alloys are usually treated with sandblast and acid-etch under nitrogen protection, followed by storage in isotonic saline to maintain their hydrophilicity^15^. An applicable route to hydrophilic surfaces was based on a rapid treatment with diluted aqueous sodium hydroxide solutions^16^. Long period of ultraviolet (UV) treatment creates hydrophilic surfaces and retards the attack of attached biofilms in implant surfaces^17^.

Plasma is a neutral ionized gas and is the fourth state of matter. It is comprised of particles in permanent interactions which consist of photons, electrons, positive and negative ions, atoms, free radicals and excited or non-excited molecules. Non-thermal plasma (NTP), also named cold atmospheric plasma (CAP), is acquired at lower pressures and less power with a low macroscopic temperature^18^. In this condition, most of the coupled energy is transmitted into the electrons with a higher temperature, and the neutral particles and ions support only inappreciable energy and are keep cold. NTP treatment was capable of increasing surface energy and wettability without changing the surface topography to accelerate protein adsorption and promote biological behavior of the cells^20^. Though NTP treatment, Ti implants surface with hydrophilia was obtained before surgical operation to reduce carbon contamination and avoid aging, thus enhancing new bone formation and osteointegration and reducing the time of healing procedures. Moreover, NTP treatment was significantly more effective than UV treatment on the material surfaces^21^, and showed faster surface functionalization and more extensive hydrophilization, when compared to UV treatment^22^. Prior studies suggested enhanced osseointegration through single gas plasma treatment^23^. However, concerning the effect of other gases, gas mixtures would be alternative. NTP plays an important part in introducing different functional groups. It was reported that oxygen plasma could introduce a mixture of mainly -OH functional groups, and argon plasma was used to introduce the free radicals^19^. Based on these results, the mixed gas (argon/oxygen) plasma could obtain more different functional groups, and have a much more effective influence than a single gas on the implant. It was reported that the beneficial effects of Ti surfaces came from for the argon plasma reactor for 12 min or the oxygen plasma reactor for 10–12 min^20,23^. Without any clamping devices, the Ti samples were introduced into the chamber of the apparatus more prone to contamination.

Therefore, in the present study, we developed novel apparatus and methods of NTAP treatment with a special clamping device using mixed gas (argon/oxygen) for implant activation. The objectives of this study were to: (1) investigate the surface characteristics of the novel NTAP-Ti; (2) evaluate the biological effect of NTAP-Ti on MC3T3-E1 cell adhesion, proliferation and osteogenic differentiation; (3) examine the in vivo behavior of NTAP-Ti for osseointegration in an animal model. The following hypotheses were tested: (1) Oxygen content would be greatly increased with decreased carbon contamination on the new NTAP-Ti; (2) The attachment, proliferation and osteogenic differentiation of MC3T3-E1 cells would be significantly enhanced on NTAP-Ti; (3) The osseointegration in vivo of NTAP-Ti would be substantially promoted, compared to control Ti.

## Materials and methods

### NTAP source

**Figure 1:**
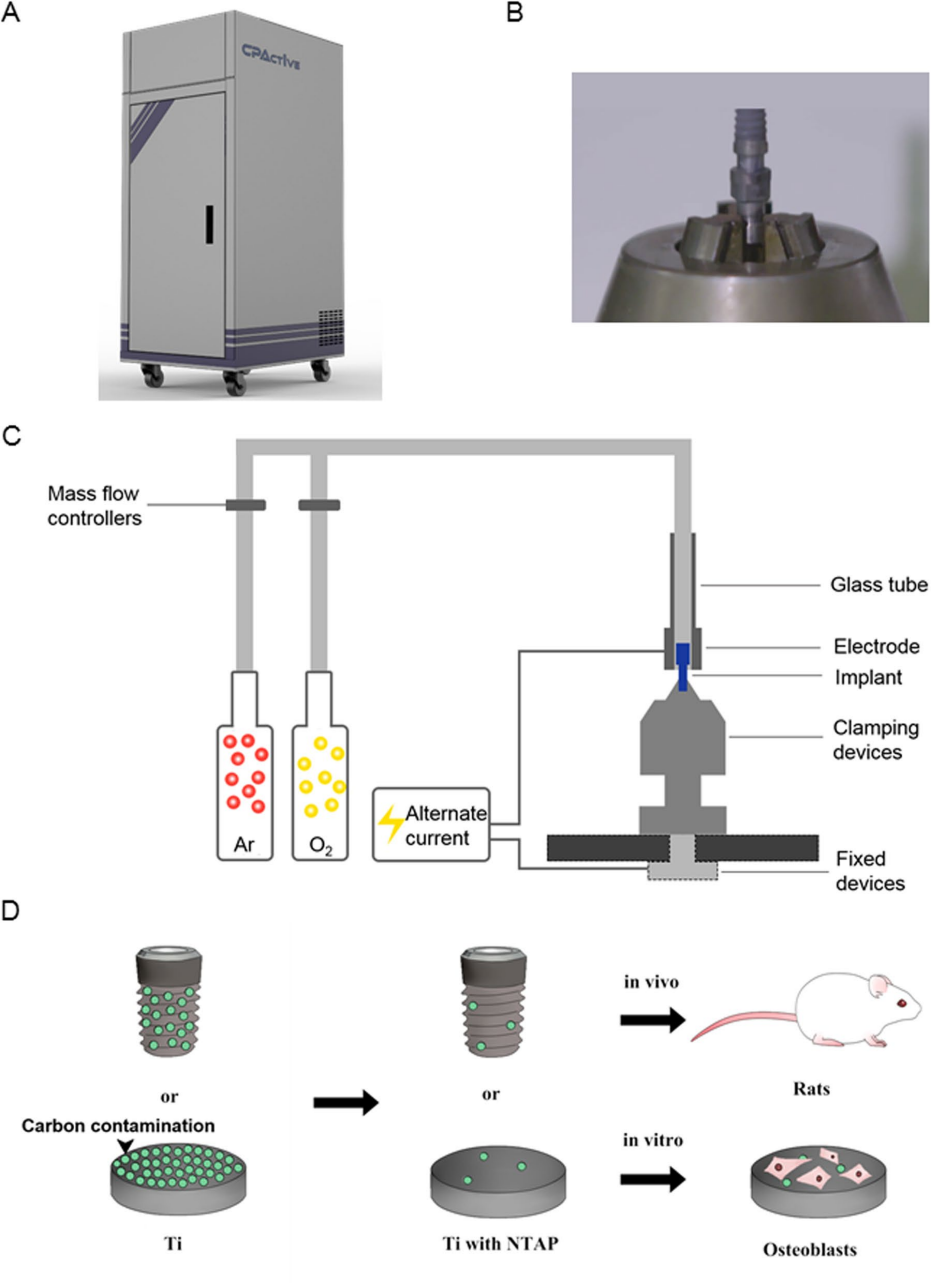
Schematic and picture of the NTAP generator. (**A**) Novel apparatus of NTAP treatment (CPActive). (**B**) The clamping devices. (**C**) The schematic diagram of the apparatus. (**D**) Schematic overview of the experimental processes.

The novel apparatus (CPActive, Chengdu, China) and methods using NTAP for implant activation were developed and described earlier (Fig. 1A)^24,25^. Briefly, the composition and flow rate of the working gases were controlled by mass flow controllers. The Ti specimen was used as an inside electrode, and an electrical field was applied to inside and outside electrodes located inside the device by an alternating current supply. The apparatus was specialized in designing of clamping devices in order to be adapted to different implant systems (Fig. 1B)^26^. This plasma source was generated by dielectric barrier discharge under the range from 20 to 50 w of electrical power, resulting in a low plasma temperature ranging from 30 to 70 °C, with the corresponding argon gas flow rate ranging from 3,000 sccm (standard cubic centimeter per minute) to 5,000 sccm and oxygen content varying from 0.1 to 1%. The schematic diagram of the apparatus was shown in Fig. 1C.

### Specimen preparation and surface characterization

Commercially pure Ti disks (grade IV, 15 mm in diameter and 2 mm in thick, Xinhangfeng Technology, China) were prepared. Ti disks were cleaned ultrasonically with absolute ethanol and distilled water for 30 min, and sterilized in autoclaves for 1 h. Cylindrical Ti implants were prepared (grade IV, 2.0 mm in diameter, 3.5 mm in length, thread pitch 0.5 mm, Wego, Shangdong, China). Ti disks and cylindrical implants were treated by NTAP for 60 s (NTAP-Ti group), as compared to control group without NTAP treatment (Ti control group) (Fig. 1D).

The surface morphologies of titanium specimens were examined using a scanning electron micrograph (SEM) (Inspect F50, FEI, USA). The surface roughness parameter was evaluated using an optical profilometer (Contour GT-K1, Veeco). The arithmetical mean surface roughness (R_a_) was measured with a measurement length of 4 mm and a cut-off value of 0.8 mm on three specimens. Wettability of the surface was determined by measuring the contact angle of a 1-μL H_2_O droplet in three different areas of each specimen surface after 3 s of dropping using a contact angle meter (JC200D2H, China). The crystalline structure of surfaces was examined by an X-ray diffraction (XRD, TTR III, Rigaku Corporation, Tokyo, Japan) at a scan rate of 2° min^−1^ over a wide range of angles (2θ = 20 − 80°). The composition and chemical state of chemical elements were measured using an X-ray photoelectron spectroscopy (XPS, Escalab 250Xi, Thermo Scientific, Madison, WI, USA), and the O1s, Ti2p, N1s and C1s peaks were obtained in the Ti surfaces^27^. All spectra were referenced to the binding energy scale of the C1s peak (284.8 eV).

### Cell culture

MC3T3-E1 mouse preosteoblasts were offered by the State Key Laboratory of Oral Diseases (Sichuan University, China). The experiments were approved by the institutional review board of Sichuan University. MC3T3-E1 cells were cultured in α-minimum essential medium (α-MEM, Gibco, Gaithersburg, MD, USA) containing 10% Fetal bovine serum (FBS, Gibco, Gaithersburg, MD, USA) and 1% Penicillin/streptomycin (PS, HyClone, Logan, UT, USA) in a humidified atmosphere of 5% CO_2_ at 37 °C. At 80% confluency, the MC3T3-E1 cells were subcultured by trypsinization (HyClone, Logan, UT, USA). The medium was replaced every 2 days. MC3T3-E1 cells were seeded on the Ti or NTAP-Ti surfaces at a density of 1.0 × 10^4^ cells/disk.

### Cell morphology and proliferation assays

After 12 h of culture, MC3T3-E1 cell morphology on Ti surfaces was observed by SEM^28^. The specimens at 12 and 24 h were stained using rhodamine phalloidin and DAPI, and examined using a fluorescence microscope (Olympus)^29^. CCK-8 assay (Dojindo) was used to evaluate the adhesion and proliferation of MC3T3-E1 cells on Ti disk surfaces at 3 h, 1 day, 3 days and 5 days^30,31^.

### Cell differentiation

After 24 h of culture, the medium was replaced with an osteogenic medium supplemented with 10 mM β-glycerol phosphate, 0.2 mM ascorbic acid and 10^–4^ mM dexamethasone for the following osteogenic-related studies. The osteogenic medium was replaced every 2 days. After 4 days, 7 days and 14 days of osteogenic differentiation, MC3T3-E1 cells were reacted with an ALP Assay Kit (Beyotime). The absorbance of solution was measured at 405 nm^32^. The MC3T3-E1 cells were stained with ARS solution (1%, pH4.2, Solarbio) and observed under a stereomicroscope (Olympus) at 7 days, 14 days and 21 days^33^. The mRNA expressions of alkaline phosphatase (ALP), osteocalcin (Ocn), osteopontin (Opn) and runt-related transcription factor 2 (Runx2) were evaluated by quantitative real-time polymerase chain reaction (qRT-PCR) using 2 × T5 Fast qPCR Mix (Tsingke)^34^. The mRNA expressions of ALP, Ocn, Opn and Runx2 were normalized on that of the reference gene β-actin. The relative mRNA expression was analyzed and calculated using the 2^−ΔΔCt^ method. The sequences of the primers used were listed in Supplementary Table S1 online.

### In vivo animal experiment and surgical procedures

The research protocol was submitted to and approved by State Key Laboratory of Oral Diseases & National Clinical Research Center for Oral Diseases conducted under the ARRIVE (Animals in Research: Reporting In Vivo Experiments) guidelines. All experiments were carried out in accordance with relevant guidelines and regulations. 4-week-old male Sprague Dawley (SD) rats (n = 70, mean weight: 100 ± 10 g) were prepared for the experiment in vivo. The animals were supported by West China Animal Experimental Center, Sichuan University, kept with free access to water and feed of balanced chow. The rats with normal appetite, activity and excretion can incorporate into the experiment.

At each surgical session, rats were anesthetized by intraperitoneal injection with 10% chloral hydrate solution (0.33 mL/100 g). The rats were in the supine position. The maxillary first molars (M1) were extracted by the probe and elevator from both sides. After 4 weeks of healing from the extraction, full-thickness flaps were elevated and cortical penetration holes were prepared for implants installation. The cylindrical Ti implants were randomly divided and inserted with or without NTAP treatment. All drilling procedures were performed under abundant normal saline irrigation and aseptic conditions. The flaps were intermittently sutured and treated by periocline ointment (2% minocycline hydrochloride ointment). Prophylactic antibiotics (80,000 units per day, Penicillin Potassium) were performed daily for 3 postoperative days^35^. Animals were sacrificed at 2, 4, and 6 weeks (w) after implantation, which were euthanized by a lethal dose injection of 10% chloral hydrate solution. Specimens were fixed in 4% paraformaldehyde and stored at 4 °C before use.

### Micro computed tomography (micro CT)

The maxillae with implant surgery region were analyzed using a μ-CT50 scanning system (Scanco Medical AG, Basserdorf, Switzerland). The tissue around implants was scanned by microCT at a high voxel resolution of 14 μm and the integration time of 500 ms with an energy level of 90 kV and 200 μA. A region of interest (ROI) was defined as the circumferential region exactly 500 μm away from the implant surface along the length surrounding the implant neck to the tip of implant. Bone parameters including the percentage of bone-to-implant contact (BIC), bone volume fraction (BV/TV), trabecular number (Tb.N), trabecular thickness (Tb.Th), trabecular separation (Tb.Sp) were measured for evaluating the microstructure of bone tissue around implant^36^.

### Histologic, histomorphometric and immunohistomorphometric analyses

Non-decalcified specimens were dehydrated in increasing grades of ethanol and embedded in epoxy resin. All non-decalcified specimens were sliced longitudinally in the buccolingual direction through the center axis of the implants and cut into 50 μm using the EXAKT diamond cutting system (EXAKT 300CP, Norderstedt, Germany) and EXAKT micro-grinding system (EXAKT 400CS, Norderstedt, Germany). All non-decalcified specimens were stained using methylene blue (MB) and basic magenta (BM) for light microscope (Leica, Germany) observation^37^.

The specimens were decalcified with 10% ethylenediaminetetraacetic acid (EDTA) for 30 days, and then the implants were removed via inverse rotation. Specimens were embedded in paraffin and prepared at 5 μm sections. Hematoxylin and eosin (HE) staining and tartrate-resistant acid phosphatase (TRAP) staining were performed^38^. Immunohistochemical evaluation of Ocn and Runx2 was conducted^30^. Stained sections were observed and photomicrographed with a light microscope (Leica, Germany). Histomorphometric analyses were performed using ImageJ software (version 1.51v, Bethesda, MD). The osteoclast number, and Ocn-positive and Runx2-positive cells were quantified directly using ImageJ software.

### Statistical analyses

All statistical analyses were performed using the IBM SPSS software (SPSS 19.0, Chicago, IL, USA). All data were expressed as means and standard deviation (SD). Significant differences of data among different groups were calculated using one-way analysis of variance (ANOVA) with the Student−Newman−Keuls test for multiple comparisons. *P* < 0.05 was considered statistically significant.

## Results

### Surface characterization

**Figure 2:**
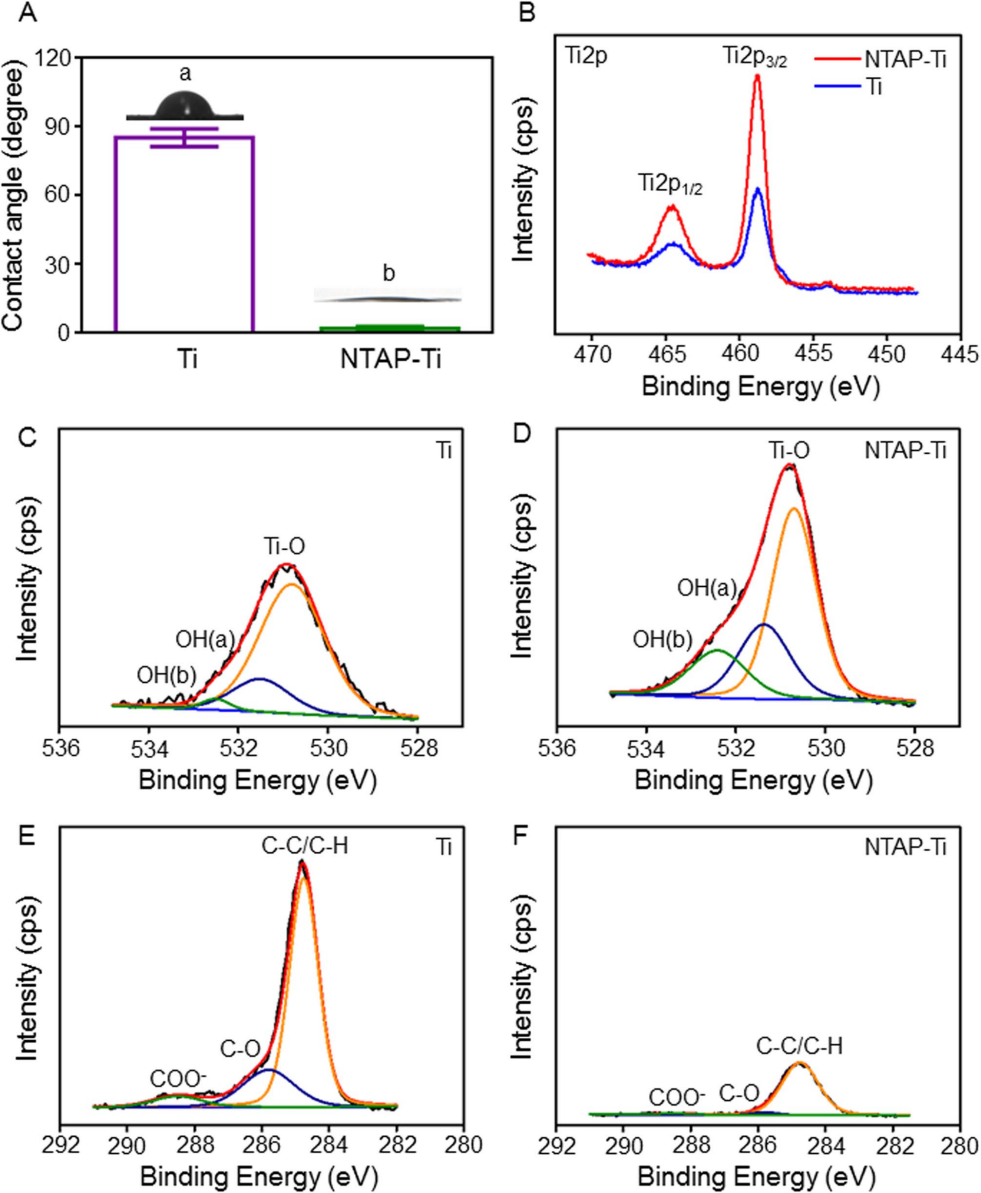
Surface characterization of NTAP-Ti in association with their biological effects. (**A**) Qualitative results of surface water contact angle, n = 6 specimens/group. (**B**) Changes in Ti2p spectra of titanium and NTAP-treated titanium surfaces. O1s spectra of titanium under XPS analyses: (**C**) Ti, (**D**) NTAP-Ti. C1s spectra of titanium: (**E**) Ti, (**F**) NTAP-Ti. Data are shown as mean ± SD. Values with dissimilar letters are significantly different (*P* < 0.05).

SEM analysis indicated that the surface microstructure of Ti specimens was similar in relatively smooth morphology with a few scratches from the polishing process (Supplementary Fig. S1A). There was no marked difference in R_a_ value between Ti control disks and NTAP-Ti disks (Supplementary Fig. S1B). R_a_ values of the control, and NTAP-Ti groups were 0.32 ± 0.03 and 0.27 ± 0.03 μm, respectively (*P* > 0.05). The water contact angle between Ti and NTAP-Ti groups had a significant difference (*P* < 0.05). The surface contact angle in NTAP-Ti group decreased by 100% than Ti group (*P* < 0.05), contributing to a superhydrophilic surface (Fig. 2A). There was no statistical difference in the crystalline structure of surfaces between Ti and NTAP-Ti groups (*P* > 0.05) (Supplementary Fig. S1C), which was consistent with the SEM analysis result.

The XPS analysis of Ti disks showed peaks of C1s, O1s, and Ti2p. The Ti2p peak was divided into doublets of the Ti2p_1/2_ and Ti2p_3/2_ peaks at binding energy of 464.5 eV and 458.7 eV, respectively. The Ti2p_1/2_ and Ti2p_3/2_ peaks revealed an increase of 60% and 100% in NTAP-Ti group compared to the control Ti group, respectively (Fig. 2B). The relative area percentages of O1s, and C1s peak components were computed in Table 1. The major peak of O1s spectra was at binding energy of 530.7 eV (Fig. 2C, D), and was divided into three components, namely Ti–O peak (530.8 eV), OH(a) for acidic Ti–OH peak (531.5 eV) and OH(b) representing basic Ti–OH group (532.5 eV). The O1s peak in NTAP-Ti group remarkably increased by 50% compared to the control Ti group, and area percentages of OH(a) and OH(b) notably promoted by 40% and 500% in NTAP-Ti specimens, respectively, illustrating enhanced surface hydroxylation. The C1s peak revealed the presence of C–C/C-H peak at 284.9 eV, C–O peak at 286.1 eV, and COO^−^ peak at 288.5 eV (Fig. 2E, F). The major peak of C1s at a binding energy of 284.8 eV markedly decreased by 80% in NTAP-Ti group compared to Ti group.

**Table 1:** The area percentages of O1s, and C1s peak components on two surfaces.

Group	O1s			C1s		
	Ti–O	OH(a)	OH(b)	C–C/C-H	C–O	COO^−^
Ti	79.34%	17.79%	2.87%	73.02%	20.99%	5.99%
NTAP-Ti	56.51%	25.45%	18.04%	93.22%	3.99%	2.79%

### Influence of NTAP treatment on the cellular morphology of MC3T3-E1 cells

**Figure 3:**
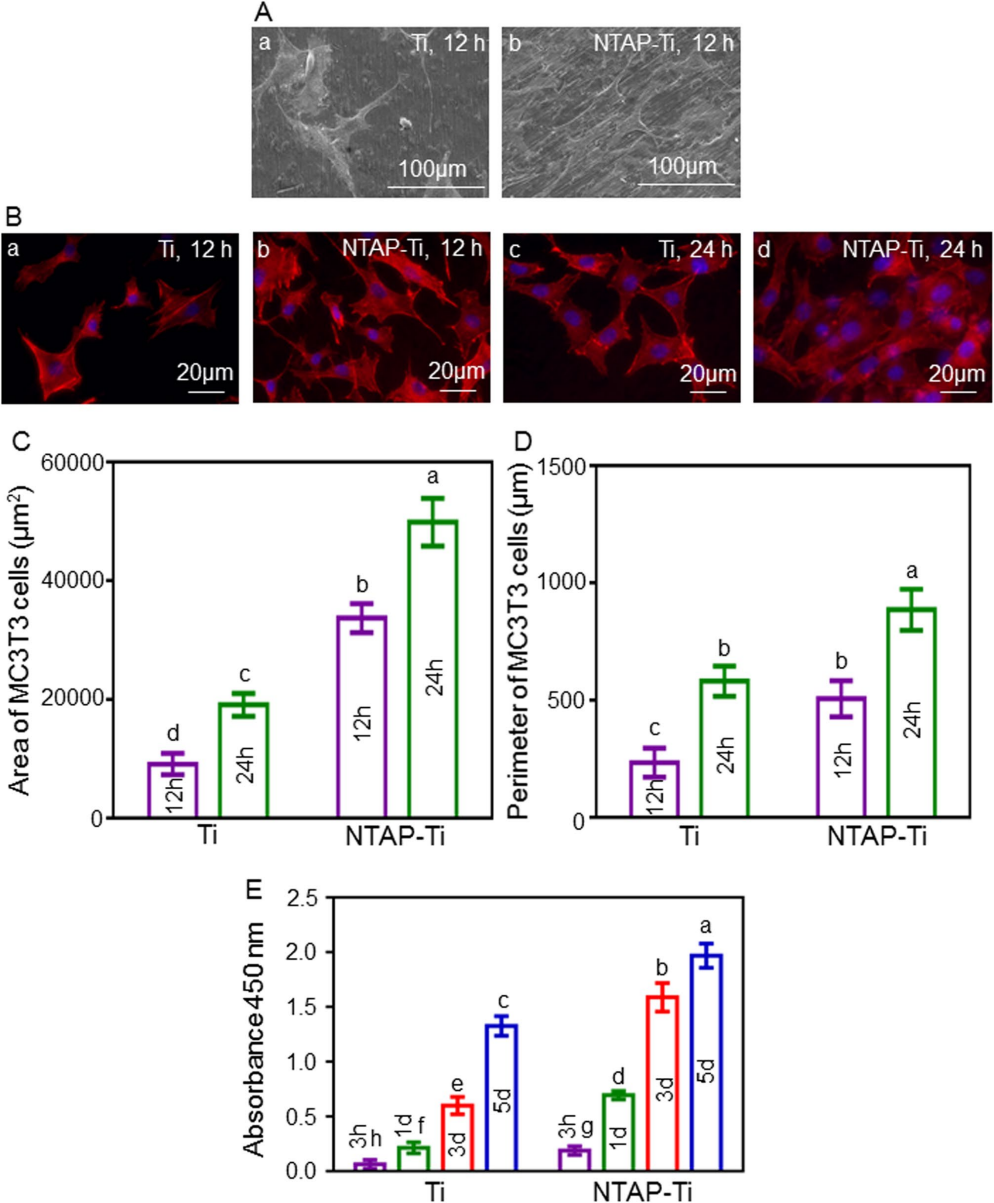
Biological effects of NTAP-Ti seeded with MC3T3-E1 cells. (**A**) SEM morphology of MC3T3-E1 cells on titanium surfaces at 12 h: (a) Ti, (b) NTAP-Ti. (**B**) Fluorescence microscopy images of MC3T3-E1 cells on titanium surfaces at 12 and 24 h: (a, c) Ti, (b, d) NTAP-Ti. Spreading area (**C**) and perimeter (**D**) of adherent MC3T3-E1 cells cultured on the titanium surfaces for 12 and 24 h. (**E**) Cell adhesion and proliferation of MC3T3-E1 cells on titanium surfaces after 3 h, 1 day, 3 days and 5 days of culture. Data are shown as mean ± SD; n = 6 specimens/group. Values with dissimilar letters are significantly different (*P* < 0.05).

There was an increased number of MC3T3-E1 cells in NTAP-Ti group over time. After 12 h of incubation, there was a great quantity of MC3T3-E1 cells in NTAP-Ti group, and the morphology of cells remained mature on the NTAP-Ti surface in better spread and extension with more protrusions (Fig. 3A, B). At 24 h, the number of polygonal cells was increased in NTAP-Ti group, and much more protrusions could be observed (Fig. 3B). As compared to the control group, the mean cell area of MC3T3-E1 cells was increased by 3 folds and 1.5 folds in NTAP-Ti group at 12 and 24 h, respectively. The perimeter of MC3T3-E1 cells was enhanced by 100% and 50% on NTAP-Ti surface after 12 and 24 h of incubation, respectively (Fig. 3C, D).

### Influence of NTAP treatment on the adhesion and proliferation of MC3T3-E1 cells

The results of CCK8 assay showed that the absorbances at 450 nm were increased over time, and the absorbances were increased by 2 folds, 2.5 folds in the early stage of incubation at 3 h and 1 day, respectively, in NTAP-Ti group than the control group (Fig. 3E). The NTAP-Ti group resulted in a 150% and 50% increase of the absorbances at 450 nm, when compared to the control Ti group at 3 and 5 days, respectively. The assay indicated a higher initial adhesion and proliferation of MC3T3-E1 cells on NTAP-Ti surface, suggesting that the NTAP-Ti was biocompatible and had no adverse effects on the cell health.

### Influence of NTAP treatment on the osteogenic differentiation of MC3T3-E1 cells

**Figure 4:**
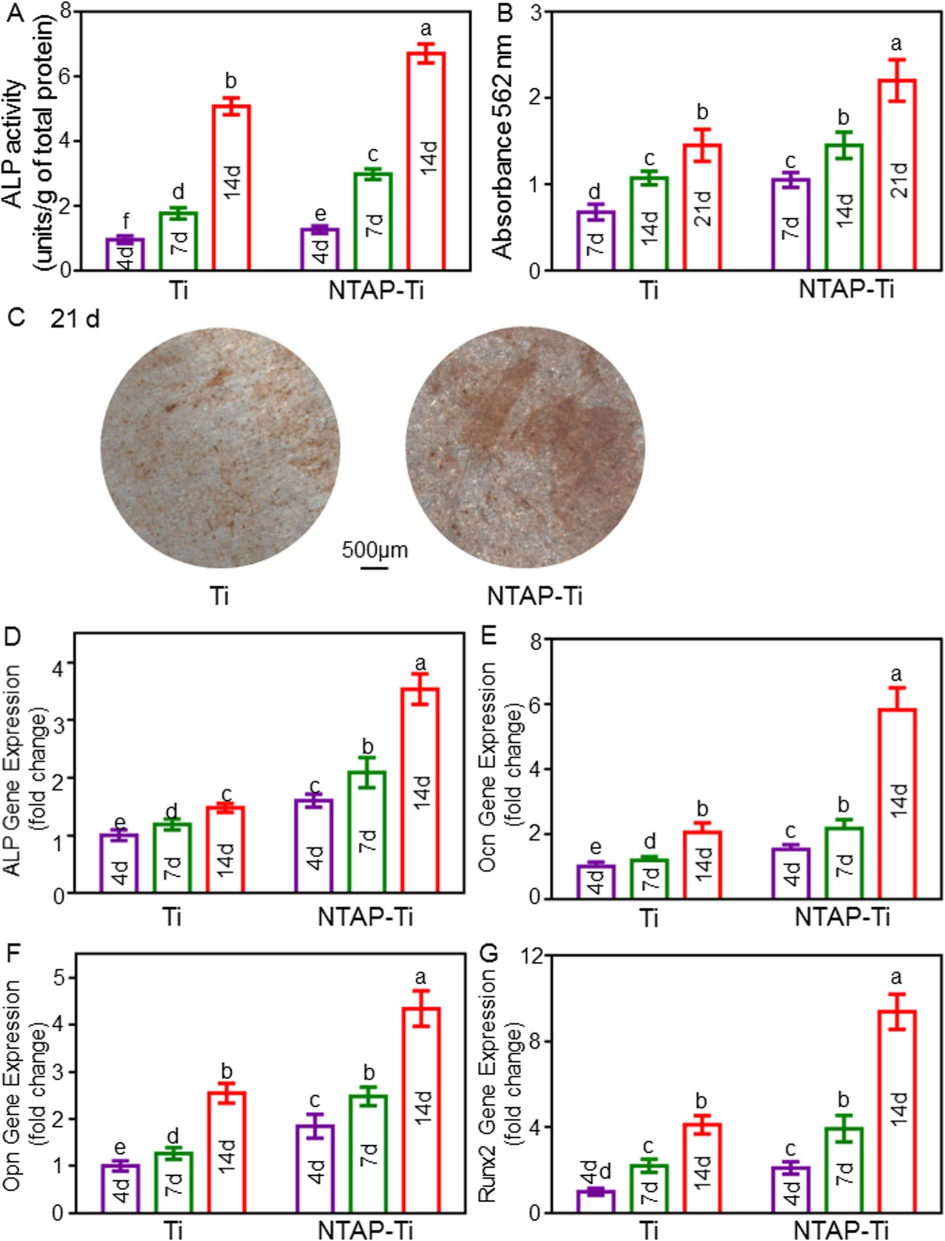
Enhanced osteogenic differentiation and mineralization of MC3T3-E1 cells on NTAP-Ti. (**A**) Colorimetric quantitative ALP activity of MC3T3-E1 cells cultured on titanium surfaces for 4, 7, and 14 d. (**B**) Colorimetric quantitative results of calcification, n = 6 specimens/group. (**C**) Alizarin red S (ARS) staining of MC3T3-E1 cells cultured on titanium surfaces for 21 days. expression of osteogenic-related genes of MC3T3-E1 cells cultured on titanium surfaces for 4, 7, and 14 days: (**D**) ALP, (**E**) Ocn, (**F**) Opn, (**G**) Runx2. Data are shown as mean ± SD; n = 6 specimens/group. Values with dissimilar letters are significantly different (*P* < 0.05).

The ALP activity revealed an increase with increasing culture time. After 4, 7 and 14 days of osteogenic differentiation, NTAP-Ti group had higher (*P* < 0.05) ALP activity by 30%, 70%, and 30% than that on control group, respectively (Fig. 4A). The ALP activity exhibited a remarkable increase of 4 folds from 4 to 14 days on the NTAP-Ti surface (*P* < 0.05).

Alizarin red S staining verified the formation of mineral synthesis after 7, 14 and 21 days of osteogenic differentiation. Mineral synthesis by MC3T3-E1 cells was increased from 7 to 21 days. By 21 days, the dark red staining of minerals synthesized and accumulated on the surfaces of NTAP-Ti disks (Fig. 4C). The absorbances at 462 nm, namely the extracellular mineral synthesis generated by MC3T3-E1 cells, were enhanced by 50%, 35%, and 50% (*P* < 0.05) in NTAP-Ti groups as compared to the control Ti group at 7, 14 and 21 days, respectively (Fig. 4B). The mineralized nodules and extracellular calcium deposits of NTAP-Ti surface displayed an increase of 100% from 7 to 21 days (*P* < 0.05).

The expressions of osteogenic genes (ALP, Ocn, Opn, and Runx2) were measured using qRT-PCR. After 4, 7 and 14 days of osteogenic differentiation, NTAP-Ti group resulted in 60%, 75% and 150% increases of ALP expression when compared to the control Ti groups, respectively (*P* < 0.05) (Fig. 4D). The expression of Ocn was strengthened by 50%, 80% and 180% (*P* < 0.05) in NTAP-Ti group compared to the control group at 4, 7 and 14 days, respectively (Fig. 4E). The expression of Opn on NTAP-Ti surfaces was 80%, 100%, and 70% higher than that of the control at 4, 7 and 14 days (*P* < 0.05) (Fig. 4F). The Runx2 expression showed an increase of 100%, 80% and 150% in NTAP-Ti group at 4, 7 and 14 days (*P* < 0.05) (Fig. 4G). The ALP expression increased rapidly at the early stage, and the Ocn, Opn, and Runx2 expressions were sharply increased during the late stage, of the osteogenic differentiation process.

### NTAP-enhanced in vivo integration with bone

**Figure 5:**
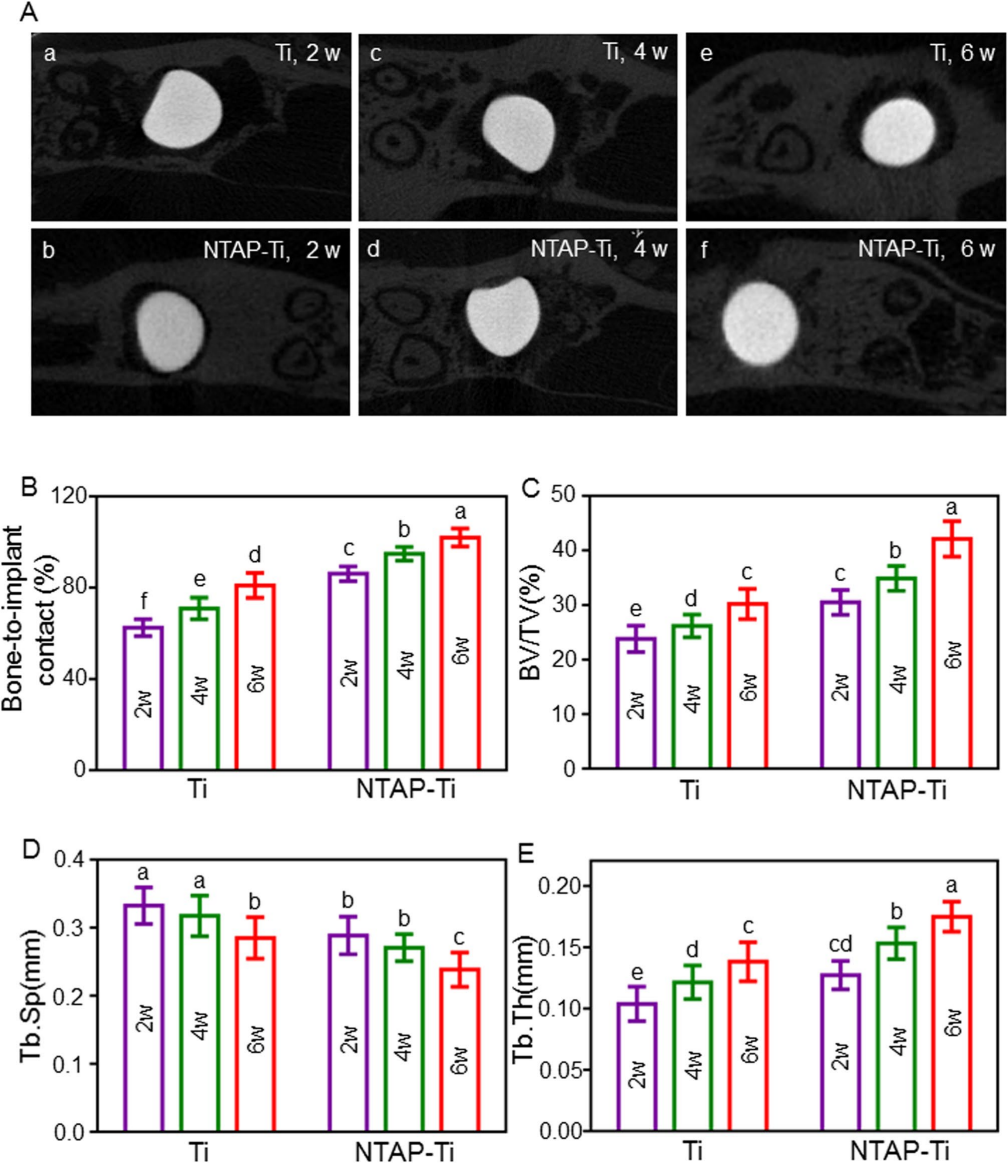
Micro-CT analysis of implant regions after 2, 4 and 6 w of healing in vivo. (**A**) Representative transverse micro CT images at the center of implant for 2w (a, b), 4w (c, d), and 6w (e, f). (**B**) Bone–implant contact percentage (BIC) value after the healing period of 2, 4, and 6 w. (**C**) Bone volume fraction (BV/TV). (**D**) Trabecular thickness (Tb.Th). (**E**) Trabecular separation (Tb.Sp). Data are shown as mean ± SD; n = 6 specimens/group. Values with dissimilar letters are significantly different (*P* < 0.05).

The M1 extraction rat model was established, and the osseointegration of implants was examined in vivo. Representative transverse micro CT images at the center of implant were shown in Fig. 5A. The bone–implant contact percentage (BIC) was evaluated with the linear percentage of direct bone-to-implant contact to the total surface of implant. At the 2, 4, and 6 w postoperation, the BIC value of the NTAP-Ti group was higher by 40%, 30%, and 25% (*P* < 0.05), when compared to the control Ti group, respectively (Fig. 5B). The BIC after the healing period of 4 w approached 100% in NTAP-Ti group. The BV/TV value demonstrated a significant increase of 28%, 35% and 40% in NTAP-Ti group in contrast to Ti group after 2, 4, and 6 w of healing, respectively (*P* < 0.05) (Fig. 5C). At each time point, there was no significant difference of Tb.N value between NTAP-Ti group and Ti group (*P* > 0.05). As compared to the control Ti group, the Tb.Sp value showed a marked reduction in NTAP-Ti group at 2, 4, and 6 w, respectively (*P* < 0.05) (Fig. 5D). NTAP-Ti group revealed an 20%, 25% and 26% increase of Tb.Th when compared to Ti group at 2, 4, and 6 w, respectively (*P* < 0.05) (Fig. 5E). The increased BV /TV value, accompanied by increased Tb.Th and decreased Tb.Sp in NTAP-Ti group represented superior new bone formation around the implants.

**Figure 6:**
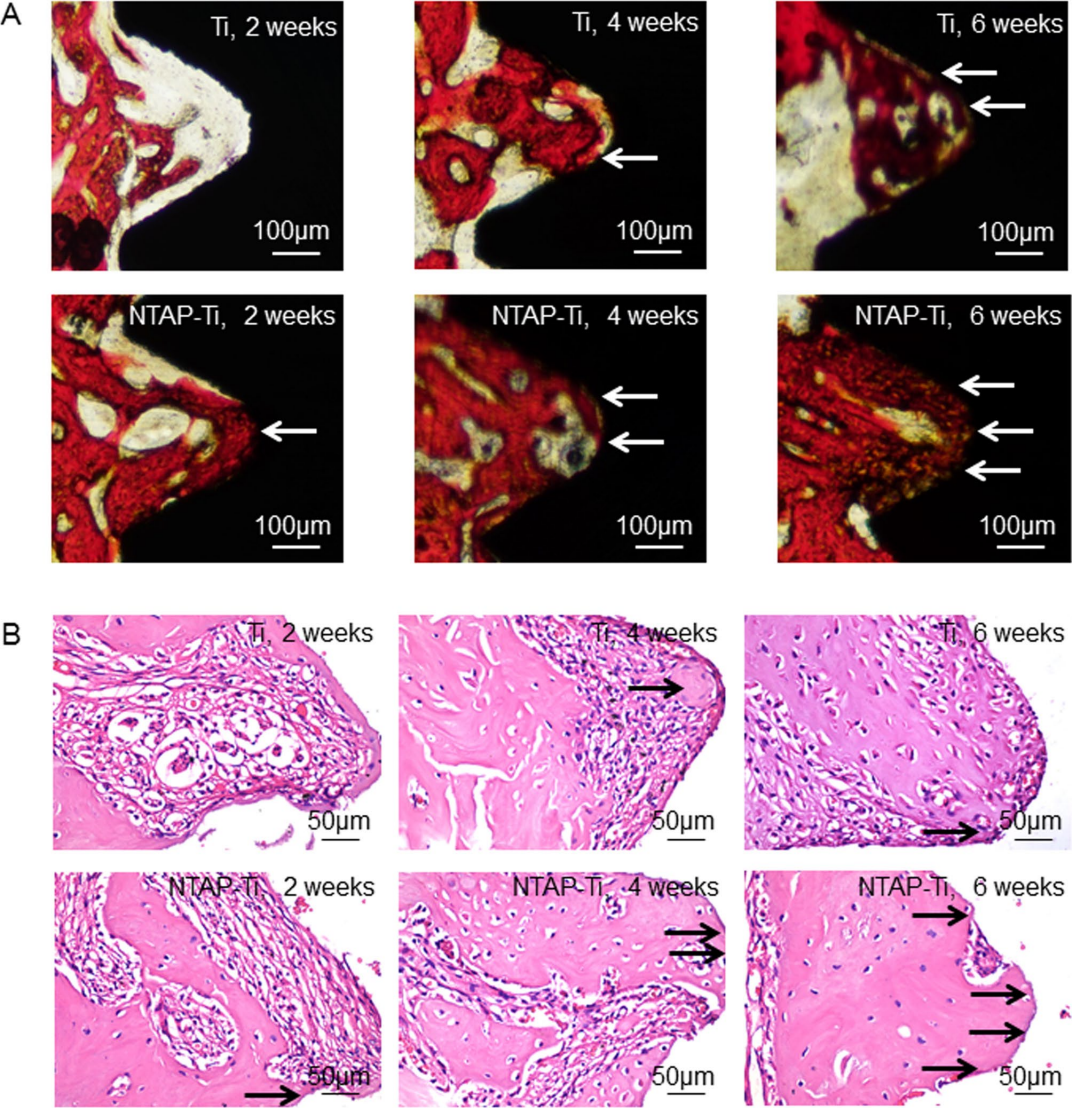
Histology and morphology of implant regions in rats. (**A**) Ground sections illustrating the healing after 2, 4, and 6 w. (**B**) Hematoxylin and eosin (HE) staining after implantation of 2, 4, and 6 w. There was more new bone with increasing time from 2 to 4 w around the implants. New bone formation (arrows) was mainly observed in NTAP-Ti group at each implantation period.

After 2 w of healing, the distribution of new bone was different between Ti and NTAP-Ti implants. The new bone mainly occupied in the area relatively distant from the implant surfaces in Ti group. A thin layer of new bone was formed in contact with the implant surface, without any penetration of soft tissues between the implant and the bone in NTAP-Ti group. More new bone was formed and thickened with more contact with the surface of the implant at the bone-implant interface after healing for 4 and 6 w in the NTAP-Ti group (Fig. 6A, B).

**Figure 7:**
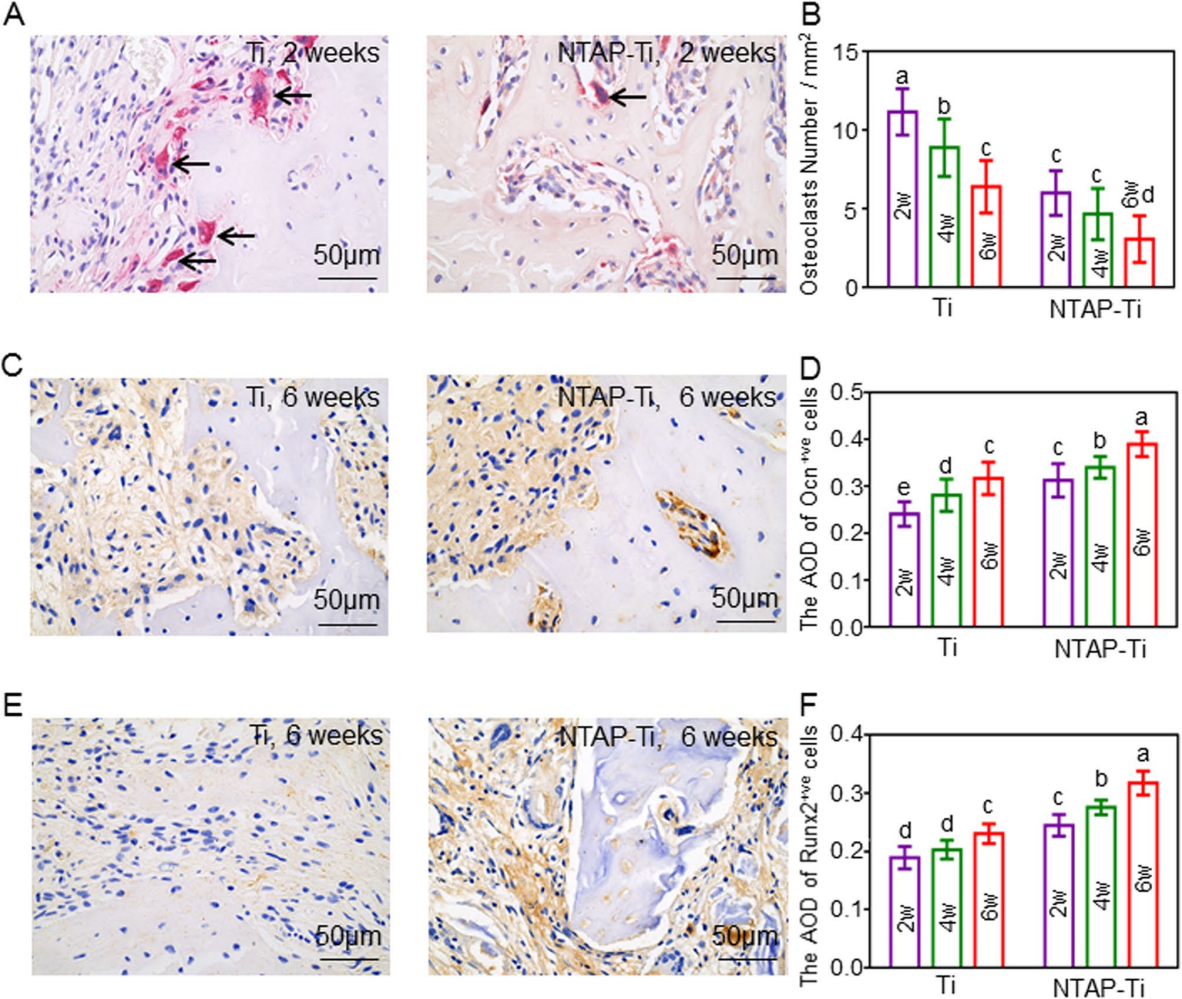
TRAP and Immunohistochemical staining, and histomorphometry analysis of implant regions in rats. (**A**) TRAP staining for 2 w of Ti and NTAP-Ti. Osteoclasts (arrows) were located in the resorption lacunae of the alveolar bone surface around implant. (**B**) Quantitative results of osteoclast number. Immunohistochemistry for Ocn (**C**) and Runx2 (**E**) at 6 w in Ti and NTAP-Ti groups. Colorimetric quantitative results of Ocn (**D**) and Runx2 (**F**) staining. Data are shown as mean ± SD; n = 6 specimens/group. Values with dissimilar letters are significantly different from each other (*P* < 0.05).

TRAP staining was performed to calculate the osteoclast number. TRAP-positive multinucleated were located in the alveolar bone surface around implant (Fig. 7A). The number of osteoclasts decreased by 40% and 50% in Ti and NTAP-Ti groups from 2 to 6 w, respectively. The osteoclast number showed a decrease of 50% in NTAP-Ti group compared to the Ti group at 2, 4 and 6 w post-operation (*P* < 0.05), suggesting a reduction in bone resorption (Fig. 7B). The expressions of Ocn (Fig. 7C) and Runx2 (Fig. 7E) were evaluated in the osseointegration studies. The average optical density (AOD) of Ocn revealed increases of 20%, 30% and 23% in NTAP-Ti group as compared to Ti group after implantation for 2, 4 and 6 w, respectively (*P* < 0.05) (Fig. 7D). The AOD of Runx2 was increased by 30%. 35% and 40% in NTAP-Ti group when compared to the Ti group at 2, 4 and 6 w, respectively (*P* < 0.05) (Fig. 7F). Higher expressions of Ocn and Runx2 were determined in the NTAP-Ti group, further demonstrating an enhancement in bone formation.

## Discussion

Ti is a popular biomaterial for the replacement and restoration of surgical implants. However, the aging of Ti results in biological degradation of the surfaces from being bioactive to bioinert. In the present study, we designed and fabricated novel CPActive apparatus and methods for implant activation using NTAP to improve the physical and biological of Ti surfaces and promote the in vivo osseointegration in an animal model. The mixed gas (argon/oxygen) plasma was used to introduce different functional groups on Ti surfaces. A special clamping device of the universal apparatus was designed to be appropriate for different Ti specimens and avoid contamination during which samples were introduced into the chamber of the apparatus for the first time.

Generally, organic impurities from the atmosphere attach to Ti surfaces over time, resulting in an increase in surface hydrophobicity^39,40^. A single oxygen plasma could greatly enhance the content of -OH than others, and the gas impact of argon plasma was helpful to introduce free radicals. Both of them have an ability to reduce carbon content. In the present study, argon/oxygen plasma was aimed to achieve good surface properties. The results of surface characterization demonstrated that the NTAP treatment did not remarkably alter the surface structure and morphology. Notably, the NTAP-Ti group resulted in contact angles of zero degrees. Meanwhile, the XPS results also revealed decreased C–C/C-H peak in the NTAP-Ti group, whereas OH(a) and OH(b) peaks increased. The NTAP treatment in CPActive was fast and high-efficient. This apparatus and methods changed the hydrophobic Ti surface into a superhydrophilic surface with less carbon contamination. The positive effects of Ti surfaces immediately using CPActive for 60 s were comparable to those immediately after other plasms treatment for 10–12 min.

The previous studies showed that Ti with higher surface wettability improved the spreading and growth of cells in vitro^41^. Furthermore, it was reported that hydrophilic surfaces accelerated the initial cell processes of attachment and proliferation of mesenchymal stem cells (MSCs)^42^. The process of osseointegration is based on the adhesion and proliferation of pre-osteoblasts and the differentiation of these cells into osteoblasts on implant surface. In the present study, the MC3T3-E1 cells was chose to investigate the biological behavior and osteogenic differentiation of pre-osteoblasts cells on NTAP. The NTAP-Ti enhanced the MC3T3-E1 cell attachment and proliferation. MC3T3-E1 cells had a polygonal shape and attached well with much more protrusions in the NTAP-Ti group, while some cells with short filopodia were observed in the control Ti group. These results indicated that NTAP-Ti disks accelerated the level of cytoskeleton development, further promoting the adhesion, spread, and proliferation of the osteoblasts.

The osteoblastic differentiation of MSCs and osteoblasts was enhanced on hydrophilic surfaces^43,44^. Prior studies suggested the gap of the ALP level between plasma-treated and untreated Ti surfaces narrowed in early time^45^. In the present study, the level of ALP activity was higher in NTAP-Ti group at 7 days of culture compared to Ti group. Moreover, the NTAP-Ti surface increased the formation of calcium nodules and the generation of extracellular calcium deposits. ALP, Ocn and Opn are considered differentiation markers of the osteoblastic phenotype and expressed in cells of the osteoblastic lineage in vivo and in vitro^46–48^ Runx2 is an essential transcription factor for osteoblast differentiation during the early stage of osteoblast differentiation and the stage of bone maintenance^49^. The ALP and Ocn expression exhibited a rapid increase of 150% and 180% in NTAP-Ti group at 14 days, respectively. NTAP-Ti surface exhibited a 100% increase of Opn expression levels at 7 days and a 100% and 150% increase of Runx2 expression at 4 days and 14 days. Therefore, the novel NTAP-Ti achieved much better osteogenic differentiation and mineralization of MC3T3-E1 cells.

Some previous studies proved that plasma could enhance osseointegration with approximately 45% BIC in 6 w^50^. In the present study, results from micro CT figured out that increased BIC, BV/TV, Tb.Th values and decreased Tb.Sp around NTAP-Ti implant indicated superior mineralized tissues in NTAP-Ti group. The BIC value was up to virtually 100% in NTAP-Ti implant after 4 w of healing. Furthermore, new bone formation was clearly observed in direct contact with the NTAP-Ti implants surfaces. TRAP is as a marker of osteoclasts and bone resorption^51^. NTAP-Ti had increased number of Ocn-positive and Runx2-positive cells and decreased number of osteoclasts, indicating the increased osteogenic and inhibited osteoclast activities of NTAP-Ti surfaces. Therefore, the novel NTAP modification in CPActive facilitated the differentiation of osteoblasts and bone regeneration, and suppressed the bone resorption, simultaneously.

The initial bone reconstitution and successful osseointegration promoted the long-term stability of implants^52,53^. Surface modification techniques, such as electro-erosion, sandblasting, polishing, acid-etching and machine-tooling, were demonstrated to be economic and efficacious approaches^54–56^. Moreover, it is common to use rats as animal models for medical research, and it is necessary to use larger animals to further simulate biological behavior under more complicated conditions^57,58^. Furthermore, it was reported that the ALP, Ocn, Opn, and Runx2 could be activated by the Wnt pathway^59–61^. In the present study, the increased expression of ALP, Ocn, Opn, and Runx2 revealed that the Wnt signaling pathway may be activated by NTAP treatment to accelerate the process of bone remodeling, which needs us further demonstration. The novel and universal apparatus and methods of NTAP for implant activation with a special clamping device are promising to enhance the surface hydrophilic property and strengthen the osteogenic performance of Ti implants, as well as to shorten the healing time and improve the success rate of implantation.

Taken together, the novel NTAP-Ti accelerated the physical and biological effects and enhanced the integration with bone. The novel and universal apparatus (CPActive) and methods with a special clamping device using gas mixtures are promising for implant activation by high-efficient changing Ti to a hydrophilic surface to greatly improve dental and orthopedic applications.

## Supplementary information

Supplementary information.
