# Supplementary material for: Effects of novel non-thermal atmospheric plasma treatment of titanium on physical and biological improvements and in vivo osseointegration in rats
Source: Sci Rep. 2020 Jun 30;10:10637. doi: 10.1038/s41598-020-67678-z (PMC7327023; doi:10.1038/s41598-020-67678-z)

**Supplementary Information**

**Effects of novel non-thermal atmospheric plasma** **treatment of titanium on physical and biological improvements and *in vivo*** osseointegration **in rats**

Zheng Zheng ^a, b^, Xiaogang Ao ^a, b^, Peng Xie ^a, b^, Jie Wu ^c^, Yuqing Dong ^c^, Deping Yu ^c^,

Jian Wang ^a, b^, Zhimin Zhu ^a, b^, Hockin H. K. Xu ^d-f^, Wenchuan Chen ^a, b, *^

^a^ State Key Laboratory of Oral Diseases & National Clinical Research Center for Oral Diseases, West China Hospital of Stomatology, Sichuan University, Chengdu, China;

^b^ Department of Oral Prosthodontics, West China Hospital of Stomatology, Sichuan University, Chengdu, Sichuan, China;

^c^ School of Mechanical Engineering, Sichuan University, Chengdu, China;

^d^ Biomaterials & Tissue Engineering Division, Department of Advanced Oral Sciences and Therapeutics, University of Maryland Dental School, Baltimore, MD 21201, USA;

^e^ Center for Stem Cell Biology and Regenerative Medicine, University of Maryland School of Medicine, Baltimore, MD 21201, USA;

^f^ University of Maryland Marlene and Stewart Greenebaum Cancer Center, University of Maryland School of Medicine, Baltimore, MD 21201, USA

For: *Scientific Reports*

(Submitted in April 2020)

**^∗^ Corresponding author:** Prof. Wenchuan Chen, email: [hxkqcwc@scu.edu.cn](mailto:hxkqcwc@scu.edu.cn).

Supplementary Table S1. The sequences of the primers used for qRT-PCR.

| **Gene** | **Primer sequences (F, forward; R, reverse)** |
| --- | --- |
| **β-actin F** | AGATTACTGCTCTGGCTCCTAGC |
| **β-actin R** | ACTCATCGTACTCCTGCTTGCT |
| **ALP F** | CTGCCTGAAACAGAAAGTCTGC |
| **ALP R** | TATGTCTTTACCAGGAGGCGTG |
| **Ocn F** | GGACCATCTTTCTGCTCACTCTG |
| **Ocn R** | ACCTTATTGCCCTCCTGCTTG |
| **Opn F** | TTCTCCTGGCTGAATTCTGAGG |
| **Opn R** | GCTGCCAGAATCAGTCACTTTC |
| **Runx2 F** | ACGAAAAATTAACGCCAGTCGG |
| **Runx2 R** | CACTTCACCCTCAGGACCG |

**
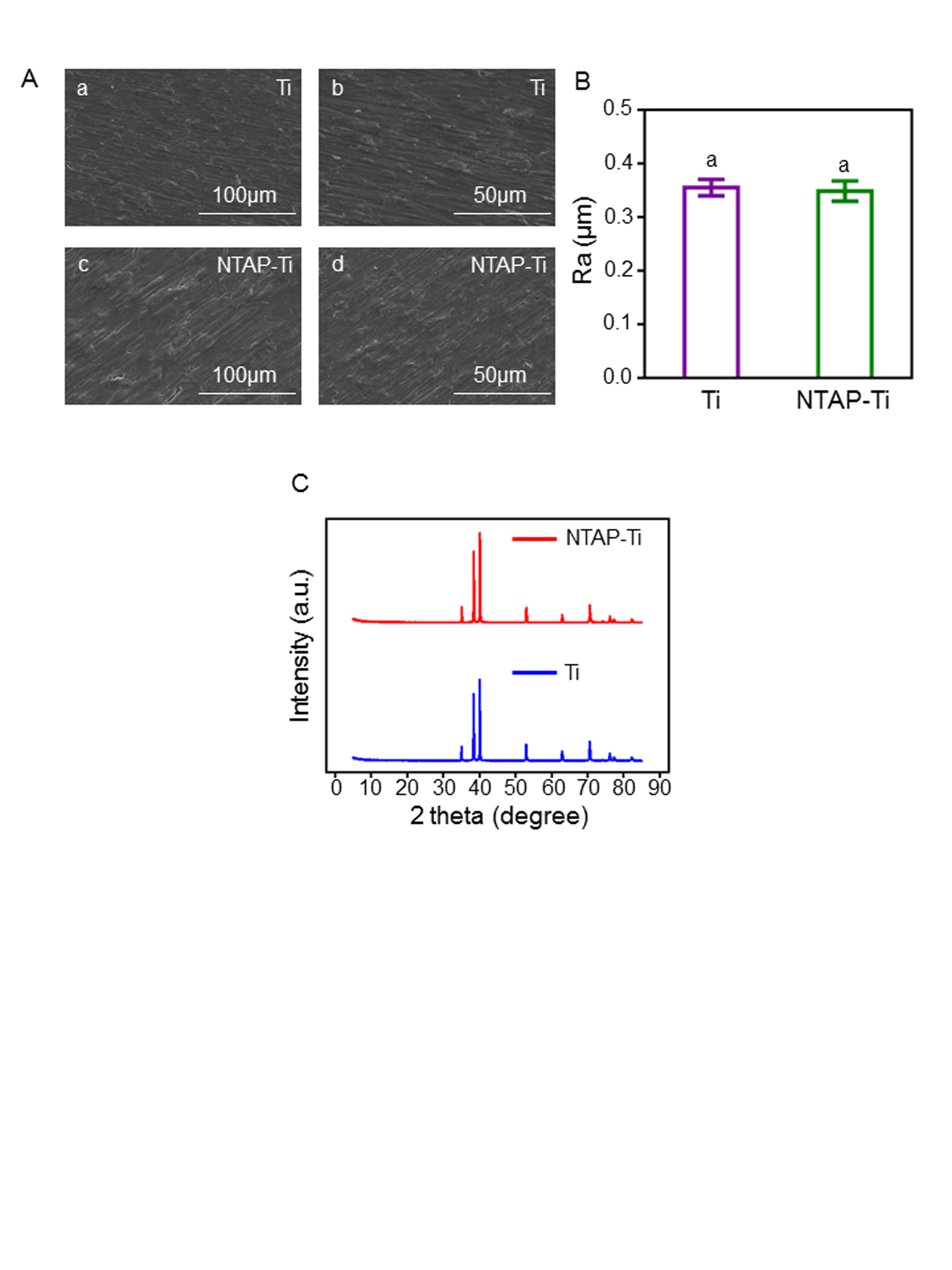
**

Supplementary Fig. S1. (A) FE-SEM images of different specimens. (B) The arithmetical mean surface roughness values of titanium specimens. (D) X-ray diffraction (XRD) spectrum of titanium specimens. n = 3 specimens/group. Data are shown as mean ± SD. Values with dissimilar letters are significantly different (p < 0.05).

**Completed copy of the ARRIVE checklist**


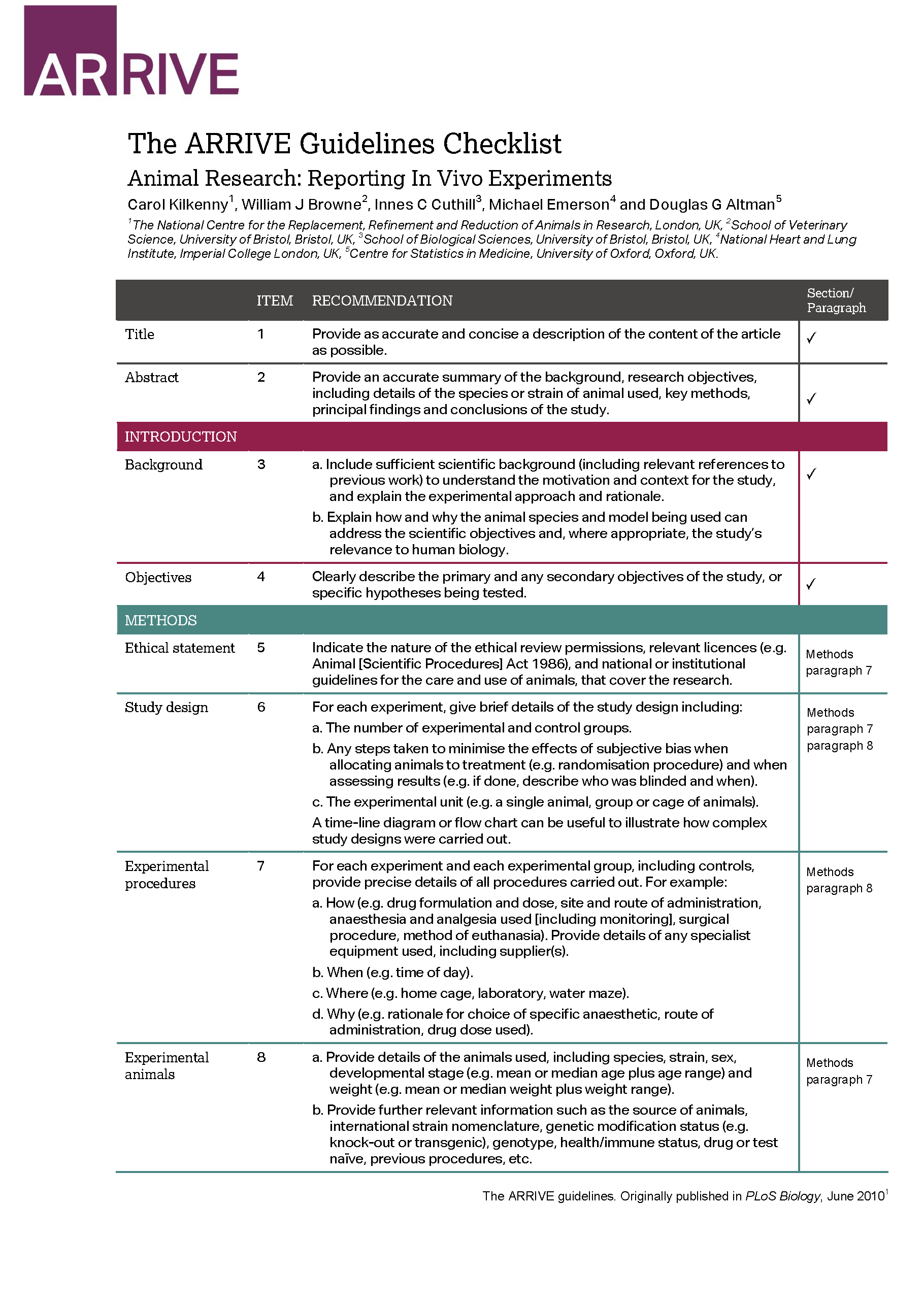

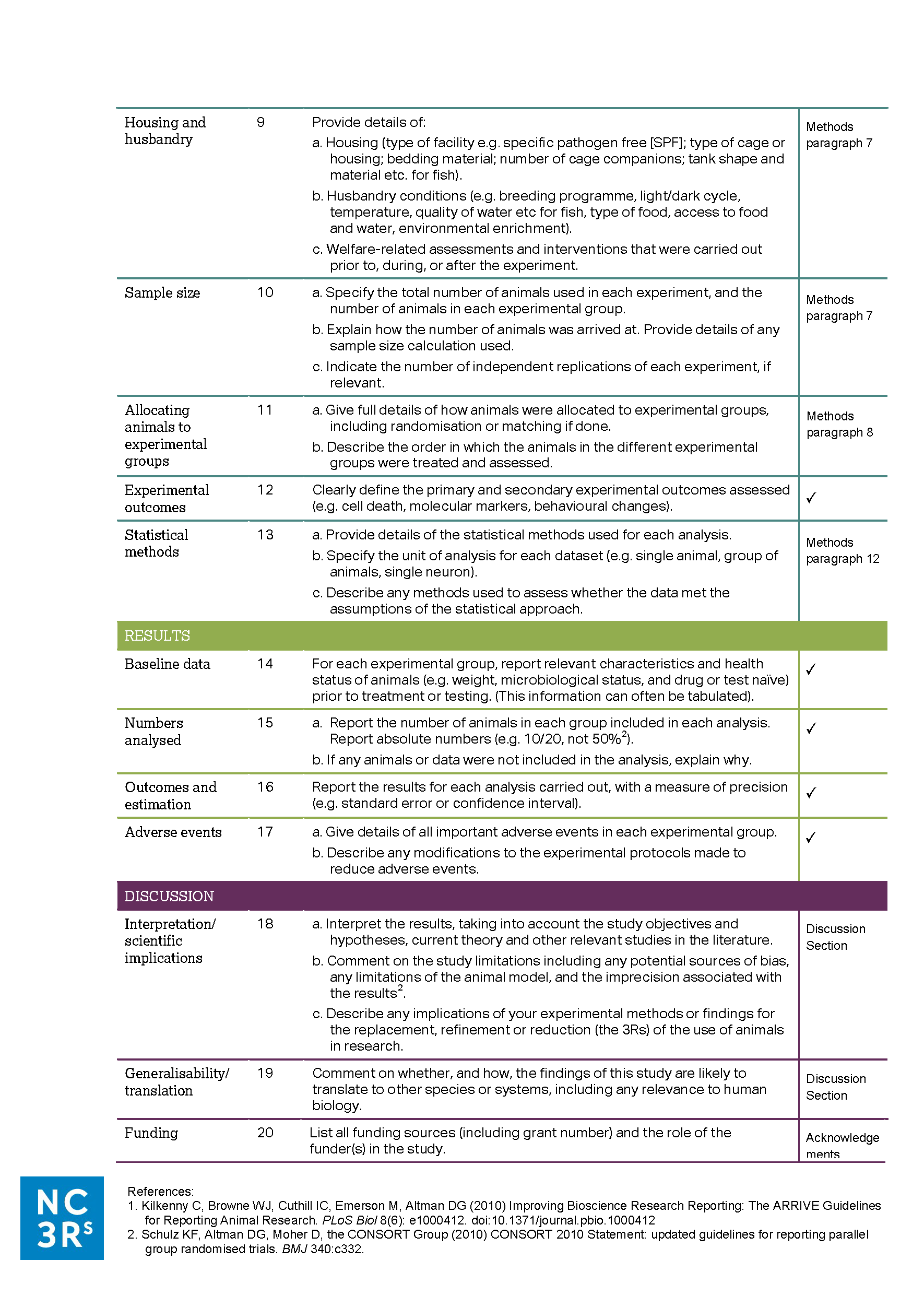

Supplement: Supplementary file 1 — Supplementary information. [file 41598_2020_67678_MOESM1_ESM.doc]
